# 3D deformation field in growing plant roots reveals both mechanical and biological responses to axial mechanical forces

**DOI:** 10.1093/jxb/erw320

**Published:** 2016-09-24

**Authors:** François Bizet, A. Glyn Bengough, Irène Hummel, Marie-Béatrice Bogeat-Triboulot, Lionel X. Dupuy

**Affiliations:** ^1^UMR EEF, INRA, Université de Lorraine, 54280 Champenoux, France; ^2^James Hutton Institute, Ecological Sciences group, Dundee DD2 5DA, UK; ^3^School of Science and Engineering, University of Dundee, Dundee DD1 4HN, UK

**Keywords:** 3D imaging, biomechanics, buckling, kinematics, root growth, Young’s elastic modulus.

## Abstract

Strong regions and physical barriers in soils may slow root elongation, leading to reduced water and nutrient uptake and decreased yield. In this study, the biomechanical responses of roots to axial mechanical forces were assessed by combining 3D live imaging, kinematics and a novel mechanical sensor. This system quantified Young’s elastic modulus of intact poplar roots (32MPa), a rapid <0.2 mN touch-elongation sensitivity, and the critical elongation force applied by growing roots that resulted in bending. Kinematic analysis revealed a multiphase bio-mechanical response of elongation rate and curvature in 3D. Measured critical elongation force was accurately predicted from an Euler buckling model, indicating that no biologically mediated accommodation to mechanical forces influenced bending during this short period of time. Force applied by growing roots increased more than 15-fold when buckling was prevented by lateral bracing of the root. The junction between the growing and the mature zones was identified as a zone of mechanical weakness that seemed critical to the bending process. This work identified key limiting factors for root growth and buckling under mechanical constraints. The findings are relevant to crop and soil sciences, and advance our understanding of root growth in heterogeneous structured soils.

## Highlight

Maximal axial pressures exerted by freely growing roots are restricted by root buckling but are increased when root lateral bracing is provided.

## Introduction

Plants are largely sessile organisms that rely on their root systems for anchorage and for the exploitation of water and nutrients from soil. Plants deploy complex root architectures to acquire soil resources efficiently, but penetration of soil is a great mechanical challenge. Roots are made of relatively soft tissues, and they must overcome considerable mechanical resistance from the soil to elongate or initiate new root primordia. Roots growing in hard soil show a severe reduction in growth rate, an increase in root diameter and various modifications of cell anatomy and morphology (Atwell, 1993; Bengough *et al.*, 2006; Lipiec *et al.*, 2012). The root system architecture is modified, with reduced root length distribution being observed, in regions where the soil is compacted (Unger and Kaspar, 1994).

How individual root apices overcome physical obstacles such as large soil particles, aggregates or compacted soil layers is still poorly understood. Avoidance strategy whereby a root exploits paths of least resistance such as cracks or soil pores is an essential component of plant adaptation to hard soils. However, root growth in channels can still result in radial stress in root tissues, with root elongation depending on the channel tortuosity, on the root angle, and on the magnitude of circumnutation movements (Kolb *et al.*, 2012). Traffic of modern agricultural machinery may create compacted soil layers where few continuous macropore channels exist (Lipiec *et al.*, 2012; Valentine *et al.*, 2012). In such soils, root elongation depends on the lubrication of the root apex to limit frictional forces and on the generation of sufficient growth forces to overcome the soil resistance to deformation (Azam *et al.*, 2013).

The process of cell elongation is central to root growth and to root penetration in hard soil. Cell elongation is sensitive to different environmental signals such as temperature and water availability (Pahlavaian and Silk, 1988; Yamaguchi *et al.*, 2010). Within a root, cell elongation only occurs in the first few millimeters of the apex. Primary growth is mainly axial and tissues are distributed symmetrically around the root longitudinal axis. Along the root apex, the relative elongation rate (or elemental elongation rate; EER) follows a dissymmetric bell-shaped pattern as revealed by kinematic studies (Peters and Baskin, 2006). Stress-induced modifications of EER patterns have been mainly analysed in steady states (Beemster and Baskin, 1998; van der Weele *et al.*, 2000; Spollen *et al.*, 2008; Silk and Bogeat-Triboulot, 2014), but transient growth patterns following stress onset have not often been studied. In particular the response of EER to external mechanical cues is unknown.

Powerful theoretical frameworks for kinematic analysis of growth were developed in the late 1970s (Erickson, 1976; Erickson and Silk, 1980), and a series of technological innovations have been built on these foundations. Early work relied on artificial markers and photographic film to track and quantify tissue deformation. Imaging systems available now have greatly improved with the use of high resolution digital cameras, fluorescence or infrared imaging, and the development of software that recognizes textures from tissues (particle image velocimetry; Basu *et al.*, 2007; Iwamoto *et al.*, 2013). It is striking that understanding of the biomechanics of root elongation has not progressed at a comparable rate. Early work from Lockhart (1965) proposed that cell walls deform in a viscoplastic fashion and cell elongation depends on the level of the cell wall extensibility. This has formed the theoretical basis for biomechanical studies of plant cell growth for the past decades. Lockhart’s equations were regularly augmented and expanded into growth equations giving the possibility of moving to the tissue scale (Ortega, 2010; Dyson *et al.*, 2014). In soil, root growth requires turgor pressure to create mechanical forces and overcome both the stretching of cell wall and the deformation of the surrounding soil. A remaining experimental limitation is the difficulty of characterizing the mechanical properties of living tissues *in situ*.

In this study, we present the first 3D live *in situ* biomechanical analysis of root tissues and root responses to axial mechanical forces. A new experimental system based on poplar cuttings grown in a compact hydroponic growth chamber was developed. It combined 3D stereoscopic imaging, a new optomechanical sensor and a suite of image analysis algorithms for high resolution tracking of forces and tissue deformation. The system was used to provide a detailed mechanical analysis of root responses to axial mechanical forces.

## Materials and methods

### Growth conditions

Cuttings of a commercial hybrid poplar (*Populus deltoides*×*P. nigra*, cv ‘Soligo’) were grown in hydroponics in a 4-l container (20×30×10cm) using a modified half-strength Hoagland nutrient solution supplemented with 0.8mM KH_2_PO_4_ and adjusted to pH 5.8 (Merret *et al.*, 2010; Bizet *et al.*, 2015). The solution was aerated by bubbling to prevent hypoxia and renewed twice a week (temperature: 19–21 °C). Cuttings with removed buds were grown in continuous darkness so the emerging adventitious roots grew using the cutting carbohydrates. Root growth rate was thus independent of new photosynthetates, suppressing any potential dial pattern of growth rate (Ruts *et al.*, 2012). All experiments were done within a week after root emergence, much before reserves became limiting. Subsequent root growth monitoring was also done in the dark, preventing inhibition of root growth by light (Pilet and Ney, 1978).

### Time-lapse imaging

**Fig. 1.**
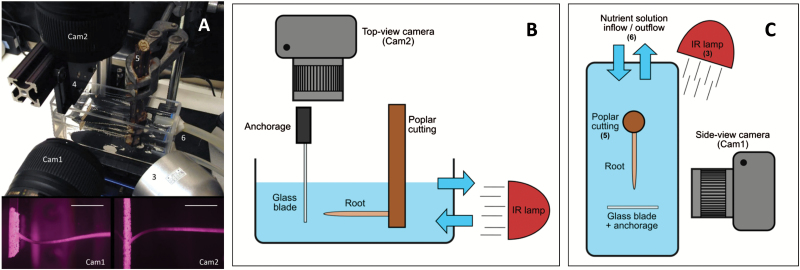
Experimental set-up used to monitor in 3D root growth and deformation. (A) Overview of the device. Cam1: side-view camera; Cam2: top-view camera; 3: infrared lamp; 4: sensor anchorage; 5: poplar cutting; 6: nutrient solution inflow and outflow. Bottom images show a poplar root after encountering the sensor and the two points of view of the buckling. Scale bars: 10mm. (B, C) Schematic lateral and top views of the experimental set-up.

For subsequent kinematics and biomechanical analysis, time-lapse imaging was performed on roots longer than 2cm (corresponding to about 3 days after root emergence). Even if several roots were suitable on the same cutting, only one root was monitored, ensuring biological replicates without statistical autocorrelation. Once a root reached the minimum required length, the cutting was transferred into a transparent Plexiglas tank filled with aerated and circulating nutrient solution for time-lapse imaging (Fig. 1). Root growth was monitored under near-infrared illumination (λ=850nm) (Bizet *et al.*, 2015). Images were taken with two defiltered cameras (Nikon D5200) mounted with a macro objective (Nikkor 60mm). The optical axis of the cameras was placed in the plane perpendicular to the direction of growth, with one camera taking images from the side and the other camera taking images from the top (Fig. 1). The parameterization and synchronization of the two cameras was carried out using the free software digiCamControl (v1.1.0). The resolution of the images was dependent on the distance between the camera and the root. The resolution was therefore 140 px mm^−1^ for the vertical camera (cam2) and 220 px mm^−1^ for the horizontal camera (cam1). We combined this experimental set-up (hydroponic tank and cameras) with a sensor dedicated to the mechanical characterization of the root.

### Sensor development

**Fig. 2.**
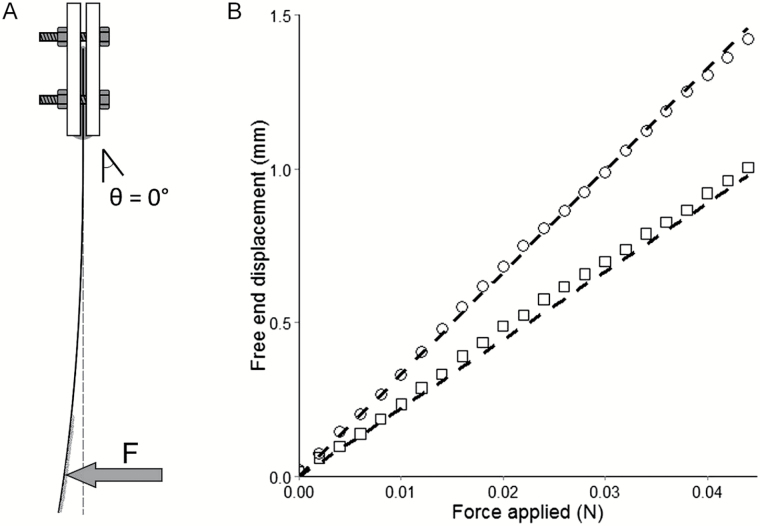
Micro-sensing of mechanical forces exerted by roots using the deflection of a glass blade. (A) The glass blade is anchored at one end and an axial force (*F*) is applied at the free end. The free end of the glass blade is coated with fine sand to prevent the root from slipping. (B) Relationship between the glass blade free end displacement and the force applied. Mechanical forces were calibrated by force application at 35mm (squares) and 40mm (circles) from the glass blade anchorage. Dashed lines indicate expected values using beam theory formulas (see text).

A sensor dedicated to the measurement of axial forces was designed in-house so that measurements could be made *in situ* (submersible) and be compatible with root EER measurement. The sensor was constructed based on the deformation of a cantilever beam. The sensor was composed of a thin glass blade (24×50mm coverslip, VWR international No. 1) clamped between a stiff aluminum base (Thorlabs BA2 post base) and a custom-made aluminum plate (57×50×2mm). For the deformable part of the sensor, glass was used since it is a ductile material with near ideal elastic properties. The mechanical behavior of glass is unchanged in water, and a glass coverslip provides a cheap and highly standardized material for measurements of high repeatability. Plastic sealing tape (Terostat VII, Teroson) was set between the two aluminum plates to apply uniform pressure on the glass blade without breaking it. In order to prevent slipping of the root, fine sand was coated on the free end of the glass blade using epoxy glue as adhesive. The force was determined based on the deflection of the glass blade measured from the images captured during the experiment (Fig. 2A).

As a first step, the sensor required calibration. Young’s elastic modulus of the sensor deformable part (the glass blade) was measured using a three-point bending test. The glass blade was placed on two roller supports. The middle of the glass blade was then displaced at a constant rate of 1mm min^−1^ by the load cell of a mechanical testing machine (Instron 5966 with a 10N load cell accurate to 25 mN at maximum load) and the force *F* was measured in reaction to the deflection of the glass blade. Young’s elastic modulus of the sensor glass blade (*E*_s_) was determined using beam theory formula for beams of rectangular cross section:

$$E_{\text{s}}=\frac{FL^{3}}{48ID}$$ (1)

with

$$I=\frac{WH^{3}}{12}$$

where *F* (N) is the force applied on the glass blade, *L* (m) is the length between the two roller supports, *I* (m^4^) is the moment of inertia, *D* (m) is the displacement of the beam at the point where the force is applied, and *W* (24mm) and *H* (0.14mm) are respectively the width and thickness of the glass blade.

When the glass blade is anchored at one end, the relation between the force applied and the displacement of the free end of the bslade is given by the formula for the deformation of a cantilever beam:

$$F=\frac{3IE_{\text{s}}D}{L^{3}}$$ (2)

where *E*_s_ (Pa) is Young’s elastic modulus of the sensor glass blade, *I* (m^4^) is the moment of inertia*, D* (m) is the displacement of the glass blade free end and *L* (m) is the length between the glass blade anchorage and the position where the force is applied.

In a second step, the sensor was tested against the cantilever beam theory using a cantilever beam bending test. The free end of the glass blade was displaced at a constant rate of 1mm min^−1^ and the force *F* was measured in reaction to the displacement imposed by the load cell of the mechanical testing machine (Instron 5966 with a 10N load cell). This measured force was compared with the force calculated from the displacement of the free end of the glass blade (Eqn 2). The sensor was tested for *L*=35mm and *L*=40mm (Fig. 2B). During experiments involving plant roots, both *D* and *L* were determined on images recorded by the side-view camera. In these experiments, roots touched the sensor at the middle of the glass blade width, preventing its torsion, at a position where 35 mm<L<40mm.

### Mechanical characterization of poplar root

The characterization of the mechanical properties of the root tissue was carried out by applying increasing axial forces on the root. The sensor was moved towards the root by increments of 0.1mm using a linear manual stage at a rate much greater than the root elongation rate. The experiment was performed until the maximum axial force the root could withstand had been recorded. This corresponds to the beginning of root buckling, i.e. a sudden structural failure characterized by a variation of the root shape in a direction different from the axial compression force. Each mechanical test was carried out within a few minutes so that movements due to growth were negligible. The rate of application of the force was also slow enough that inertia and viscous forces could be neglected. The critical Euler buckling force (*F*_crit_) was then used to determine Young’s elastic modulus of the root (*E*_r_). *E*_r_ was determined using Euler’s buckling model for a beam encastred at one end (secured with no translation nor rotation) and pivoted at the other end (rotation allowed but not translation), calculated as:

$$E_{\text{r}}=\frac{F_{\text{crit}}{\left(0.7L_{\text{r}}\right)}^{2}}{\pi^{2}I}$$ (3)

with 0.7×*L*_r_ the effective buckling length of the root and *I*=*πϕ*^4^/64. *F*_crit_ is the maximal axial force the root applied on the sensor before buckling, *L*_r_ is the root length measured as the distance between the surface of the cutting and the root tip, and *ϕ* is the root diameter. Since the diameter varied along the root apex, we measured the diameter at the end of the growing zone. *E*_r_ was determined as a function of *F*_crit_, *L*_r_, and *ϕ* using nonlinear regression analysis (Bates and Watts, 1988).

### Critical elongation force of growing roots

In order to analyse the maximum force an unconstrained root can apply on the sensor, termed critical elongation force (*G*_crit_), cuttings were placed in the hydroponic growth chamber at 3cm from the sensor. A root was placed so that its path was perpendicular to the sensor and images of the growing root were captured every minute. Experiments were stopped once the root had buckled under the pressure exerted on the sensor, generally after a few hours, depending on the length of the root and on the root elongation rate. The displacement of the sensor was used to calculate the axial force applied by the root (Fig. 1B). Critical elongation force values were then compared with critical Euler buckling force (*F*_crit_) values predicted by Eqn 3 using the mean value of Young’s elastic modulus of the root *E*_r_ previously determined in another batch of roots. *G*_crit_ was determined on six roots.

In order to test whether Euler buckling was the main physical mechanism determining the critical elongation force (*G*_crit_), the experimental set-up was modified to reduce the ability of the root to buckle. In this setting, roots grew inside a thin needle (external diameter: 1.6mm) that prevented the root from buckling. The needle was opened on one side to avoid hypoxia. The sensor was placed directly at the end of the needle and axial forces were monitored until *G*_crit_ was reached.

### Three-dimensional growth and curvature measurement

**Fig. 3.**
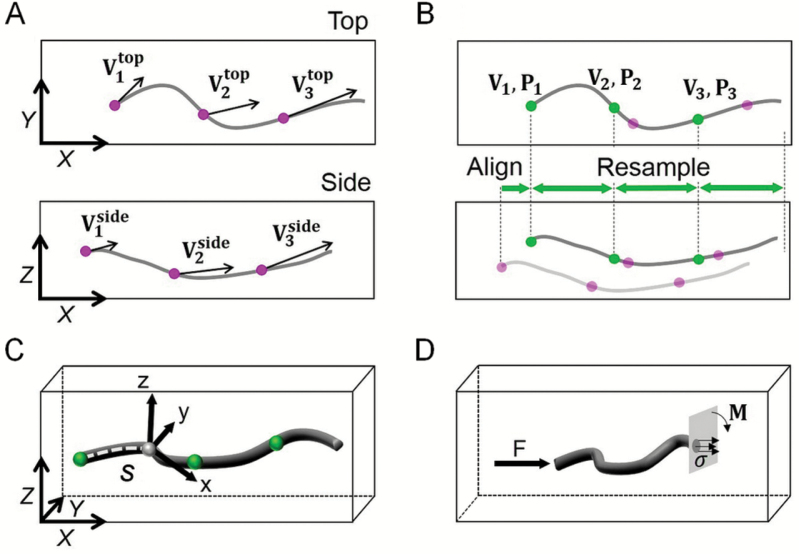
Image processing pipeline for 3D mapping of mechanical stress and elongation rate in root. (A) Top and side images of the root were analysed using particle image velocimetry. (B) Both velocity and coordinates, (respectively V and P were aligned in the direction of growth (X axis) and resampled using interpolation functions. (C) A local coordinate system was used to project full 3D velocity and coordinates in a reference coordinate system. (D) Beam theory was then used to compute stresses in the root from the force measured using the sensor.

Top and side images were captured every minute for 4–6h and were processed to reconstruct a skeleton of the root in three dimensions (*x*, *y*, *z*). Kinematics analyses were then conducted to determine the velocity and deformation fields along the root (see Erickson and Silk, 1980; Silk 1992 for details). Based on image quality from both side and top views, kinematics analyses were conducted on a selection of four out of the six roots followed for *G*_crit_ assessment. Profiles of elemental elongation rate and curvature were determined using one in every six images, i.e. calculations every 6min. The image processing pipeline involved four key steps:

Step 1, velocimetry: top and side image sequences were analysed independently using particle image velocimetry. This analysis provided the two-dimensional velocity of points regularly distributed along the root (Fig. 3A). For each point *P*_*i*_ with coordinates $\left(x_{i}^{\text{side}},z_{i}^{\text{side}}\right)$ and $\left(x_{i}^{\text{top}},y_{i}^{\text{top}}\right)$ along the root, kinematic analysis provides velocities $V_{i}^{\text{side}}$ and $V_{i}^{\text{top}}$ such that:

$$V_{i}^{side}=\left(\dot{X}\left(x_{i}^{\text{side}},z_{i}^{\text{side}}\right),\dot{Z}\left(x_{i}^{\text{side}},z_{i}^{\text{side}}\right)\right)$$

$$V_{i}^{top}=\left(\dot{X}\left(x_{i}^{\text{top}},y_{i}^{\text{top}}\right),\dot{Y}\left(x_{i}^{\text{top}},y_{i}^{\text{top}}\right)\right)$$ (4)

$\dot{X}$ and $\dot{Z}$ are the velocities along the *X* and *Z* axes on the side images, and $\dot{X}$ and $\dot{Y}$ are the velocities along the *X* and *Y* axes on the top images. The analysis was run with typically 30 points so that 1≤*i*≤30. The first point marked the quiescent center, and the last point was located within the mature zone.

Step 2, alignment and 3D resampling: the references from both views were aligned so that the spatial coordinates from the top view matched that of the side view. This was achieved by setting in both views the quiescent center as the center of the coordinate system. Thus the coordinates of the *P*_*i*_ points sampled along the root shared the same origin for top view and side view. However for *i*>1, there was no one to one matching between the coordinates from top view and from side view, preventing a direct determination of the full 3D velocity. Therefore, we used cubic spline interpolators $\hat{V}$ as a function of the coordinates along the *X* axis to match the velocities of the side view with those of the top view, $V^{\text{top}}=(\hat{V_{x}},\hat{V_{y}})$ with $\hat{V_{x}}=\;\dot{X}\left(x^{\text{side}},z^{\text{side}}\right)$ (Fig. 3B). The 3D velocity field **V** was therefore expressed as:

$$V_{i}^{}=\left(\dot{X}\left(x_{i}^{\text{side}},z_{i}^{\text{side}}\right),\hat{V_{y}}\left(x_{i}^{\text{side}},z_{i}^{\text{side}}\right),\dot{Z}\left(x_{i}^{\text{side}},z_{i}^{\text{side}}\right)\right)$$ (5)

Step 3, velocity in the root reference frame: 3D velocities were expressed in a local curvilinear coordinate system (**x,y,z**) that was linked to the geometry of the root and for which the coordinates of the points along the root were defined by the arc length *s* (Fig. 3C). The local coordinate system was defined as follow:

$$x=\frac{L_{i}}{\left\|L_{i}\right\|}$$

$$y=\;\frac{\left(0,0,1\right)\times x}{\left\|\left(0,0,1\right)\times x\right\|}$$

z=x×y

Here *L*_*i*_=(*X*_*i*+1_–*X*_*i*_, *Y*_*i*+1_–*Y*_*i*_, *Z*_*i*+1_–*Z*_*i*_) was the vector defining the direction of the root longitudinal axis between points *P*_*i*_ and *P*_*i+*1_. 3D velocities were then projected along the local root coordinate system to obtain the axial, radial and tangential components of the velocity field.

Step 4, elemental elongation rate and curvature: elemental elongation rate was obtained using three-point finite different approximation of the spatial derivative (Erickson, 1976). Root curvature κ along the root axis was calculated by assuming that consecutive root segments had small angles:

$$\kappa_{i}=\frac{1}{\left\|L_{i}\right\|}\sin^{-1}\left(\frac{L_{i}\times L_{i+2}}{\left\|L_{i}\right\|\left\|L_{i+2}\right\|}\right)$$

To allow comparisons between roots showing various growth zone lengths, elemental elongation rate and curvature along the root apices were normalized relative to the initial length of the growing zone, i.e. the zone length before encountering the sensor. Zero corresponds to the quiescent center and values above 1 indicate locations within the mature zone.

Analyses were performed using custom scripts and programs written in R and Matlab (Matlab R2011b, v7.13.0.564). Particle image velocimetry analysis was carried out using Kineroot software (Basu *et al.*, 2007).

## Results

### New sensor for *in situ* measurements of mechanical forces induced by root growth

The sensor allowed forces smaller than 10^–3^ N to be measured (Fig. 2B). In biological experiments, it was operated for forces up to 0.05N. The use of a glass element for the deformable part of the sensor ensured elastic deformation and repeatability of measurements. Axial forces applied on the sensor and the free end displacement followed a near perfect linear relationship during the cantilever bending test (Fig. 2B). Measurements recorded by the 10N load sensor from the testing machine matched the predictions from beam theory. Thus the sensor was calibrated for forces applied at different distances from the anchorage and beam theory could be used reliably to determine the force applied in this range of distances.

### Critical Euler buckling force

The first series of mechanical tests was carried out by imposing an axial force at the tip of the root at a displacement rate much faster than the root growth rate, which was thus neglected. During the build-up of forces, roots did not show lateral displacement due to bending. The sensor was displaced axially until the critical Euler buckling force of the root (*F*_crit_) was attained. At this stage, the bending of the root became visible with the maximum curvature observed close to the root tip. Further displacement of the sensor anchorage induced further bending and some slipping of the root at the point of contact with the glass blade but with no significant increase of the force. Young’s elastic modulus (*E*_r_) was determined as 32±5MPa on eight poplar roots from their lengths (29.4±3.9mm), diameters (0.56±0.03mm) and *F*_crit_.

### Critical elongation force

**Fig. 4.**
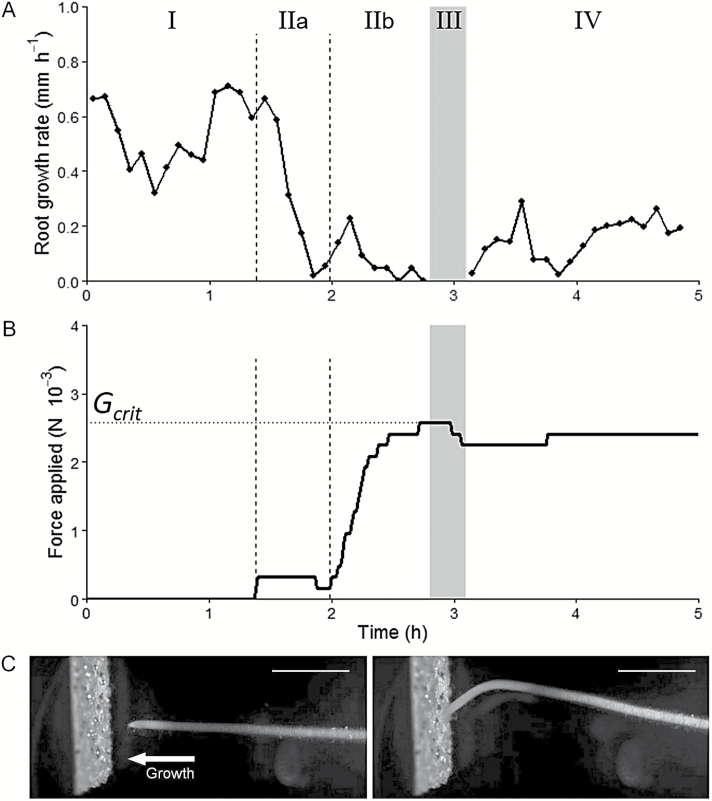
Multiphased response of a growing root while encountering the sensor. (A) Root elongation rate and (B) elongation force applied over time by a representative root pushing the glass blade. Root buckling is indicated by the grey zone and occurred once the critical elongation force (*G*_crit_) was reached. Roman numerals indicate the four different phases for subsequent analysis (see text for details). (C) Images of a poplar root seen from the side-view camera before (left) and after (right) the critical elongation force was reached. Scale bars: 10mm.

**Fig. 5.**
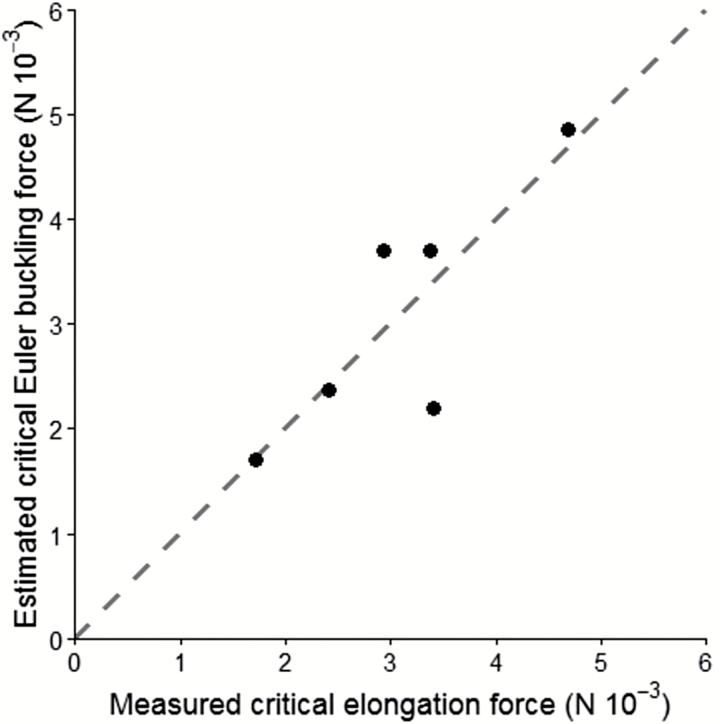
Relationship between estimated critical Euler buckling force and measured critical elongation force for growing roots. Dashed line indicates the 1:1 relationship.

The second series of experiments focused on the axial force applied by a root growing against the mechanical sensor. The critical elongation force (*G*_crit_), that is the largest force the root could apply on the sensor, was measured for six growing roots with varying elongation rates (0.51±0.12mm h^−1^) but similar diameters (0.54±0.02mm). During an initial phase the root grew without any sign of bending and the force increased continuously. When the root started bending and the force stopped increasing, *G*_crit_ was reached. Further growth of the root did not increase the force applied on the sensor (Fig. 4). For the six roots, *G*_crit_ was under 5 mN (Fig. 5) and the mean pressure was 13.3±1.1 kPa. There was no relationship between root growth rate and *G*_crit_ (*P*=0.131; data not shown). A significant linear and 1:1 correlation was found between predicted *F*_crit_ and measured *G*_crit_ (gradient: 1.01±0.09, adjusted *R*^2^=0.96, *P*<0.001; Fig. 5).

### Root response to axial mechanical forces

From the previous experiment, it was possible to describe the typical behavior of growing poplar roots encountering an axial mechanical force. The root responses could be decomposed into four phases over time (Fig. 4A, B), characterized by contrasting elemental elongation rate and root curvature: (I) before the contact with the sensor; (II) during the build-up of mechanical forces within the root, from the time the root has touched the sensor and before any root bending was observed; (III) while buckling, from the time the root started to bend (*G*_crit_ was reached) until the maximal curvature value was reached (the end of the process dominated by bending; Fig. 4C); and (IV) post-buckling growth. During phase II, roots with larger elongation rates responded to contact with the sensor in two phases. During subphase IIa, contact between the root and the sensor was established and residual movement at the root-sensor interface could be observed for a short period of time; during subphase IIb, the root tip was immobilized at the surface of the sensor (Fig. 4A, B).

**Fig. 6.**
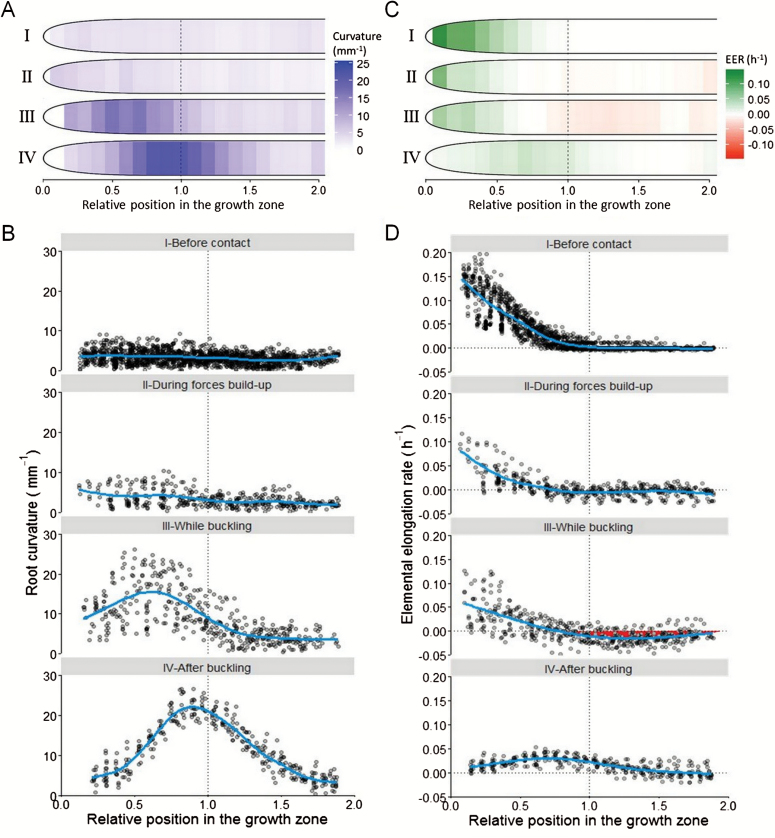
Biomechanical responses of a growing root while encountering the sensor. Temporal analyses are shown for root curvature (A, B) and for the elemental elongation rate (C, D). Roman numerals indicate the four different phases (see text for details). Positions are given relative to the initial length of the growing zone determined before contact: 0 corresponds to the quiescent center and 1 corresponds to the end of the growing zone.

During phases I and II, no significant curvature was recorded (all values <10mm^−1^, Fig. 6A, B). Curvature monitoring required capturing a large field. The subsequent reduced resolution prevented the proper determination of elemental elongation rate in the apical part of the apex (where it is very low) and led to truncated profiles of elemental elongation rate (EER). The EER was reduced by 50% and the length of the growth zone was reduced by 30% during phase II (Fig. 6C, D). At this stage, a contraction zone appeared at the beginning of the mature zone (negative EER). Tissue contraction increased up to values of −5% h^−1^ during root buckling (phase III).

During phase III, maximal bending was initially recorded in the middle of the growth zone, where the EER was large. At the end of the root buckling phase (beginning of phase IV), the maximal bending was located at the shootward border of the growth zone (Fig. 6A, B). The position of maximal bending therefore progressively moved away from the root tip, in the first few millimeters of the root. For a beam anchored at one end and having only rotation allowed at the other end, the maximum bending is predicted to take place at the middle of the buckling length of the beam (0.7×*L*_r_), at a distance of 0.35×*L*_r_. Here, mean observed location for the maximal bending point was 0.11±0.01×*L*_r_, which was much closer to the root tip than predicted from the Euler buckling theory.

After root buckling (phase IV), there was no more tissue contraction, but elemental elongation rate was strongly reduced compared with before encountering the sensor, with a maximal value of 0.05h^−1^. In addition, the growth zone length increased by 50% as compared with the initial length, i.e. before the contact with the sensor (Fig. 6C, D). During the whole experiment, no torsional movement of the root was observed.

### Impact of root buckling on the critical elongation force

When roots grow in soil, buckling is reduced because soil limits root lateral movement. Four roots were grown in needles to study the role of the resistance to root lateral movements on the critical elongation force. During this experiment, the sensor was placed directly at the end of the needle. Roots therefore applied forces on the sensor as soon as the root tip exits the needle. During the build-up of forces, the glass blade was pushed and a small fraction of the root tip was able to grow outside the needle (2.3±0.3mm). Therefore, lateral movements were restricted to the very tip of the root that grew outside of the needle. In this set-up, bending was closer to the root tip and the critical elongation force (*G*_crit_) applied was up to 15 times the one applied by freely growing roots (43.5±9.4 mN *vs* 3.0±0.5 mN). The growth rate of roots grown in needles was not significantly different from that of roots without needles (0.79±0.12 and 0.68±0.06mm h^−1^, respectively; *P*=0.46), indicating that frictional forces in the needle might be negligible. Roots grown in needles were able to apply forces on the sensor for 5–10h whereas roots grown without needles pushed the sensor for no more than an 1.5h before reaching the critical elongation force (Fig. 4A).

## Discussion

### A new system for simultaneous measurements of tissue deformations and forces

Measuring the mechanical properties of living root tissues has always been challenging. Instruments are designed for solid materials with regular geometry but biological samples are usually more complex and are made of soft matter that can be damaged when being gripped. Standard bending or tensile tests have previously been used to characterize root mechanical properties (Whiteley and Dexter, 1981; Whiteley *et al.*, 1981; Loades *et al.*, 2013, 2015). However, these tests were mainly performed on detached portions of roots in a non-growth environment, where cell turgor and mechanical stresses in root cell walls may be different from those *in situ*. Given the sensitivity of root growth to thigmo-stimulus, it is desirable to measure properties without root manipulation (Bizet *et al.*, 2015).

In this study, simultaneous *in situ* measurements of force, growth and root curvature was successfully achieved in intact growing roots with minimal modification of the growth environment. Use of near infra-red cameras allowed the exclusion of visible light from the root system, whilst the submersible sensor achieved below 10^–3^ N resolution even in the hydroponic growth solution. The distance *L* between the glass blade anchorage and the position where the force is applied will determine the sensitivity of the sensor, i.e. the minimum force required for measureable deformation of the glass blade. Greater force sensitivity and resolution may be obtained by changing the geometry or material of the deforming blade, whilst image processing algorithms determined the force exerted by the root and the rate of tissue deformation.

### Mechanical properties of poplar roots

The sensor was initially moved against the root tip to measure Young’s elastic modulus of the root (*E*_r_) at a rate of deformation much greater than the root elongation rate. Poplar adventitious roots had a mean *E*_r_ of 32MPa, which was in the expected order of magnitude for such soft tissue. In excised root apices, *E*_r_ ranged from 10 to 100MPa depending on the species considered (Dexter and Hewitt, 1978; Whiteley and Dexter, 1981; Whiteley *et al.*, 1982; Loades *et al.*, 2013). Hydroponically grown primary roots from linseed, sorghum and wheat had Young’s moduli closely comparable to our own measurements on poplar roots (*E*_r_=30–33MPa, Dexter and Hewitt, 1978; Whiteley and Dexter, 1981). Differences in age, order or diameter of roots could explain the large range of *E*_r_ among previous studies, making cross-comparisons difficult. Tissue mechanical properties will probably vary along a root due to changes in cell size and in the mechanical properties of cell walls during cell expansion. The measured *E*_r_ in our system may be characteristic of the zone most liable to initiate buckling due to its geometrical and mechanical properties, and therefore is likely to represent the lower value of Young’s modulus for poplar roots. The region presenting maximal bending was located significantly closer to the root tip than expected from an Euler buckling model, at the junction between the growth zone and the mature zone. The root diameter for the *E*_r_ calculation was measured at this location to ensure that *E*_r_ was appropriately estimated. Interestingly, this location corresponded also to a zone of tissue contraction during the build-up of the axial force before buckling, consistent with a degree of enhanced pliability (Fig. 6C, D).

The pliable zone most likely results from the tissue properties of this root section. The forces applied on a portion of root are the external pressure (here the sensor), the turgor pressure and the tension in cell walls. When the root is straight and in air or liquid medium, an external axial pressure will be transmitted uniformly along the length of the root. Tissue contraction will occur when the external load increases and is not balanced by either an increase in cell turgor pressure, or a decrease in the cell wall tension. In the rapid force application experiments, there is little time for any biological regulation of either turgor or cell wall properties. Deformation is likely to be greatest in regions of smaller turgor pressure (where the difference between turgor and wall tension is smallest) or where the combined tissue assembly of cells is more easily deformed laterally due to long thin cell geometry. Thus a pliable zone could appear where cell walls are fully extended but have not yet fully thickened, at the junction between the elongation zone and the mature zone. There, tissue has fewer cells per unit root length (as compared with more apical locations), with less and less-lignified cell wall material (as compared with more distal locations). Indeed, arrest of cell elongation is generally followed by an increase in both cell wall thickness and stiffness, whilst cessation of cell elongation is accompanied by a slight decrease of turgor pressure (0.1MPa) (Sharp *et al.*, 1990; Pritchard *et al.*, 1996; Triboulot *et al.*, 1997) that would additionally decrease the ability of the tissue to resist the external pressure in this region. The location of the zone of mechanical weakness could thus result from root cells undergoing this transition between two biological states. To our knowledge this is the first time such a zone of mechanical weakness has been reported in roots, and more detailed experiments are now required for further characterization.

### Growth and mechanics of impeded roots

In natural soils, the ability of a root to overcome a physical obstacle depends either on its ability to find a path of least resistance around the obstacle, or on the ability to generate sufficient mechanical pressure within the tissue to push its way through the obstacle. In the experiments presented here, the macroscopic roughness of the sensor surface prevented both a fast escape from the glass blade and the avoidance of it. Instead, roots responded by increasing axial forces at the tip of the root and rapidly changing the pattern of root cell expansion. The change in shape of the root appeared to be purely the result of mechanical buckling of the tissue. This situation is likely to occur in natural soils when rocks and stones have rough surface or irregular geometries, or when roots attempt to cross macropores into strong regions of soil.

Poplar roots growing in hydroponics generated a mean axial pressure of about 0.01MPa. By comparison, it has been reported that roots grown in soil may exert maximum growth pressures in the range 0.5–1MPa (Bengough *et al.*, 2011; Schmidt *et al.*, 2013). In a natural environment, the soil often provides lateral bracing to the growing root tip, making the root less liable to buckle. In addition, anchorage by root hairs in soils may also help to prevent root buckling, in situations where roots are growing within or across soil macropores (Bengough *et al.*, 2011; 2016). Buckling within the soil matrix is limited, and roots can mobilize a greater fraction of the turgor pressure to penetrate soil. We have demonstrated this effect by bracing the root into a tightly fitting needle. In this set-up, *G*_crit_ was one order of magnitude larger than for roots growing freely, generating a mean pressure close to 0.2MPa. This increase will be partly from direct shortening of the length of the root that is free to buckle (Jin *et al.*, 2013), though it should also be noted that roots may be intrinsically more sensitive to the application of axial (as compared with radial) stresses, and especially so when the root tip is unsupported radially (Bengough, 2012). Such results highlight that the presence of a physical growth medium greatly increased the capacity of roots to apply increased growth pressure and so penetrate hard soil.

Interestingly, roots clearly responded to the mechanical contact of the root tip with the glass blade. The growth rate sharply decreased within minutes of the contact, and before force build-up started (phase IIa). This suggests that roots sensed and responded biologically to very tiny forces on the root tip (<0.2 mN), before substantial mechanical resistance was encountered. After half an hour, elongation slowly recovered and the force increased until the root buckled (phase III).

The critical elongation force (*G*_crit_), that is the maximal axial force applied by the growing root before it buckled, was impressively well predicted by a model that relied solely on Young’s elastic modulus from a simple compression test (Fig. 5). The accuracy of this prediction highlights that no mechanical accommodation occurred during the short time frame of the experiment. By contrast, roots that were lastingly submitted to mechanical signals were shown to accommodate by thickening of their diameter and hardening their mechanical properties (Croser *et al.*, 2000; Tracy *et al.*, 2011). The relatively small residuals between predicted and observed values of *G*_crit_ may be due to variability of *E*_r_, lack of straightness of the root and/or inaccuracies in the measurement of root geometry. The lack of relationship between root growth rate and *G*_crit_ also supports the conclusion that root buckling during growth resulted essentially from the mechanical limit of the root tissue, rather than a biologically mediated growth response.

### Conclusion

The penetration of soil requires a root to maintain shape and a general direction of growth. The integrative approach presented here provides substantial new insights into the factors controlling root growth and deformations in response to obstacles. The root responses included several phases. Growth was first reduced by biological ‘touch’ response to very tiny forces (<0.2 mN), but was subsequently restored and axial pressure built up until mechanical buckling. The tissue at the junction between the elongation zone and the mature zone exhibited a mechanical weakness and was subjected to contraction. The maximal axial force exerted by growing roots was accurately predicted from the stiffness of tissue (measured independently) and a buckling model. Thus the maximal axial force and root growth were limited by tissue mechanical properties. Lateral support of the root increased substantially the maximal axial pressure applied by the growing root, mimicking what happens in a real soil. The challenge now is to extend experiments and theory to predict critical elongation forces in increasingly realistic, but controlled and quantifiable, soil-like environments.

## Abbreviations:

EER: elemental elongation rate
Er: Young’s elastic modulus of the root
*F*_crit_: critical Euler buckling force;
Gcrit: critical elongation force.
